# Adult Romantic Attachment and Parental Reflective Functioning in Parents of Pre-School Children: An Association Study With a Sex-Sensitive Approach

**DOI:** 10.62641/aep.v54i4.2080

**Published:** 2026-08-15

**Authors:** Leire Erkoreka, Garazi Urrutia

**Affiliations:** ^1^Galdakao-Usansolo Hospital, Osakidetza Basque Health Service, 48960 Galdakao, Spain; ^2^University of the Basque Country UPV/EHU, 48940 Leioa, Spain; ^3^Biocruces Bizkaia Health Research Institute, 48903 Barakaldo, Spain; ^4^Centro de Investigación Biomédica en Red de Salud Mental (CIBERSAM), Instituto de Salud Carlos III, 28029 Madrid, Spain

**Keywords:** object attachment, mentalization, parent-child relation

## Abstract

**Background:**

Parental reflective functioning (PRF) has important developmental roles in the offspring. The relationship between parents’ attachment style and PRF, as well as their intergenerational transmission, have been object of investigation in recent years, however, the studies have been mostly limited to mothers and conducted with semi-structured interviews. We aimed to assess this relationship using self-reports (PRF Questionnaire and Experiences in Close Relationships-Revised), and discuss their impact on child development.

**Methods:**

A total of 240 parents of 262 pre-school children were recruited via online methods. They completed both questionnaires as well as data regarding sex and age of parents and children. General linear models were conducted to assess the relationship between PRF and attachment dimensions, controlled by potential sex and age confounders.

**Results:**

Anxious attachment was significantly associated with the “prementalization” and “certainty about mental states” subscales, with the models explaining 15.8% and 7% of their variability. These associations remained constant when performing sex-disaggregated analyses, and in both cases the sex-specific models explained a greater variability in men than in women. Attachment style didn’t show any significant associations with the “interest and curiosity” subscale, nonetheless, differences between fathers and mothers in this latter dimension were also observed in the univariate analysis.

**Conclusions:**

Parental attachment styles are significantly associated with specific PRF domains, and these associations differ between mothers and fathers. Our results replicate previous findings, obtained mostly with interview-based assessments, and incorporate a sex-specific approach. Finally, the impact of PRF and adult attachment on the socio-emotional and behavioral development of the offspring is reviewed and discussed, as well as the clinical implications that could be derived from the extensive use of these tools.

## Introduction

Reflective functioning (RF) has been described as “the overt manifestation, in narrative, of an individual’s mentalizing capacity”, which is the ability to understand one’s own and others’ behavior in terms of underlying mental states and intentions [1]. Parental RF, that is, the caregiver’s capacity to reflect upon his/her own internal mental experiences as well as those of the child [2], represents a relationship-specific manifestation of the more general capacity for RF and, although they may be related, they are not identical [3].

RF is a capacity that is acquired in early childhood and in the context of attachment relationships, where parental figures, and therefore parental reflective functioning (PRF), play a leading role. PRF has been described to have other important developmental roles, such as promoting security and organization of attachment during the first year of life [1], favoring emotion regulation and the development of a sense of personal agency in the infant [4] and, even, in the parents themselves, helping them tolerate infant distress through supporting perception, interpretation, and sensitive responsiveness [5]. Another study suggests that there is an intergenerational transmission of RF, as described in relation to attachment, having found significant correlations in scores obtained by parents and offspring [6]. In the equation, however, not only parental factors such as PRF but also of the offspring come into play, establishing reciprocal influences that shape this transmission within the framework of a dyadic relationship. It has been described that maternal PRF can impact on the behavioral development and temperament of the offspring [7], but also that an infant’s temperament can act as a moderator in RF transmission [8].

As previously mentioned, mentalization is acquired within the framework of attachment relationships, so another aspect to take into account in the acquisition of mentalization in childhood, as far as it impacts on PRF, is parental attachment style. It was proposed that the parent’s attachment style acts both directly and indirectly (via PRF) on child attachment and their cognitive and socio-emotional development [3]. The same authors highlighted that the influence of the parent’s attachment style on the child’s mentalization, however, is effected via the PRF. One of the first empirical works published on the interwoven relationship established between mentalization and attachment in the intergenerational transmission of these dispositions indicated that PRF fully mediated the relationship between the attachment style of the parent and that of the child [9], they suggested that PRF is that element that remains to be defined in the transmission gap, a proposal that had previously been made on a theoretical basis [10]. A more recent meta-analysis also confirmed the relationship between PRF and child attachment, highlighting sensitivity as a partial mediator [11].

However, despite the robustness of the theoretical approach, the empirical work that has been carried out studying both the association between the parent’s attachment style and PRF, even more so when trying to add to the equation the mentalization study or the resulting attachment style in the offspring, are very scarce; this is largely due to the difficulties involved in carrying them out via extant interviews and observational measures that, until recently, have been the evaluation standard. In the aforementioned work [9], for example, all assessments were made through semi-structured interviews (Adult Attachment Interview [AAI] and Parent Development Interview [PDI]) and observational assessments (Strange Situation Procedure [SSP]). The problem with these types of evaluations is that as they are costly in terms of time, they limit sample sizes and, therefore, the representability and generalizability of the results. In this case, the number of parent-child dyads that could be studied was 40, and the results of the mediation analysis were, therefore, reported as exploratory.

A previous study, using similar methodology and measures, described that parental attachment security measured with the Attachment Script Assessment was significantly associated with PRF, measured according to the PDI, and this, in turn, with parenting behaviors [12]. Other authors observed a significant association between secure attachment measured via AAI in fathers (it was not significant in mothers) and PRF measured according to mind-mindedness, with mixed findings relating the association between this second measure and attachment security in the offspring studied by SSP [13]. However, although mind-mindedness also aims to operationalize PRF, it focuses more on the observed behavior, that is, the tendency of parents to address and accurately infer the child’s mental state during an ongoing interaction, than on remembered mutual interactions.

Self-reported tools for studying adult attachment have existed for decades, such as the Experiences in Close Relationships (ECR), which have a great consensus and extensive use. These measures, and in particular the ECR or its revised version (ECR-R), differ from the AAI both in the type of examination (self-report versus semi-structured interview) and in the attachment figure assessed. While the AAI focuses on assessing the coherence of discourse in relation to childhood attachment figures (caregivers), self-reported measures focus on assessing the quality of the relationship with significant figures, preferably romantic, in adulthood. More recently, a self-reported questionnaire was also developed that was shown to adequately assess PRF: the Parental Reflective Functioning Questionnaire (PRFQ). This questionnaire was developed taking as starting points the reflective functioning manual for the AAI, the PDI, and the Reflective Function Rating Scale. Self-reported questionnaires have the main advantage of allowing large-scale assessments, although they also have disadvantages such as the risk of lower objectivity and overlap, among others. This instrument, the first self-reported tool to assess PRF, has been validated in several languages in recent years [14, 15, 16].

A few studies have assessed the relationship of attachment in parents, measured with adult attachment self-reports, and PRF. When assessed using the PDI as a measure of PRF, the results were mixed: some authors did not observe a significant association [17], while Dollberg [18] found a quasi-significant positive relationship between PRF and anxious attachment. In the studies that used PRFQ as a measure of PRF, results have also been mixed: an association, only in mothers, between higher scores in both the anxiety and avoidance scale and higher scores in prementalizing modes has been described [4], but it has been also reported that anxious attachment is related to pre-mentalizing modes exclusively among fathers [19]. In another study in which anxiety and avoidance scores were categorized into secure and insecure attachments, this finding was replicated, observing a significant association between insecure attachment and prementalizing modes [20]. The absence of collinearity between both scales was verified, which is one of the problems when using self-reported tools. The only data published on fathers, to date, did not present any significant associations between attachment styles and PRF [4].

There are no studies, to our knowledge, on the degree of agreement between results obtained measuring PRF via PDI or PRFQ. This could be the basis of the differences seen in the degree of association between attachment and PRF when using self-reports and PDI, compared with using adult attachment self-reports and the PRFQ. These divergences occur, for example, when studying the attachment style with the AAI and adult attachment self-reports [21], however, in this case, it is understandable as it is a dynamic construct throughout life [22, 23], and to assess it at different vital moments and in relation, also, to different attachment figures. As already mentioned, the development of the PRFQ was inspired by the PDI, among other scales, however, the correlation between both measures has not been reported.

Finally, in the field of parenting, gender has a special relevance that should be transferred to research. Historically, social constructs around motherhood and fatherhood have differed greatly, with parenting being almost exclusively the domain of mothers. However, we can say that the abyss has been narrowed in recent years and the social roles associated with motherhood and fatherhood are in the process of change. Nevertheless, there are also biological differences between men and women that may impact on parenting behaviors [24, 25], thus, promoting research in the field of parenting, which includes both mothers and fathers, analyzing similarities and differences between both groups, is essential to understand in depth the weaknesses and areas for improvement in the field of parenting that can be translated into specific recommendations and interventions at clinical, social, and educational levels. Much of the work published on the relationship between parental attachment and PRF was carried out only with mothers and, in the few that also incorporated fathers, significant differences were identified regarding the values and associations observed in mothers. Differences in attachment patterns were described between mothers and fathers [26], and between the sexes in general [27]. Differences in PRF between mothers and fathers were also detected in the scarce literature published on the subject, but they were isolated data that should be replicated with new research to become conclusive.

Taking into account the scarcity of publications in this field, we decided to study the relationship between adult attachment and PRF in a sample of parents of children aged between 0 and 5 years, prioritizing achieving a large sample through the use of self-reported questionnaires, which, at the same time, allows us to expand the body of research performed with these tools. The data analysis was also carried out incorporating a sex-specific approach, aiming to determine whether there are different trends between mothers and fathers.

## Materials and Methods

### Participants and Procedures

Information about the project and the invitation to participate was disseminated through social networks and mailing lists in January and February 2022. The target audience was parents from the general population with children between 0 and 5 years old. The invitation explained the purpose and objectives of the study, and once consent was obtained for participation, the questionnaires were completed online through a link. In addition, the adults who answered the questionnaires provided both their age and sex, as well as those of the child they were thinking of when answering the PRFQ. The filling out of the questionnaires was carried out in a fully anonymized manner, making it impossible to know the identity of the participants. In those cases where they had more than one child aged between 0 and 5 years, they could complete the PRFQ for each child. The final sample comprised 240 parents (29 men, 211 women, with a mean age of 35.91 ± 4.64 years) of 262 children (140 boys, 118 girls, in 4 children the sex was not specified, with a mean age of 2.46 ± 1.52 years). The research adhered to the ethical principles set out in the Declaration of Helsinki and was evaluated by the Ethics Committee of the University of the Basque Country.

### Measures

Parental Reflective Functioning Questionnaire [4]: This is a self-reported questionnaire consisting of 18 items, which are answered using a 7-point Likert scale (1 = strongly disagree to 7 = strongly agree). It explores reflective function, which is the ability to understand or interpret the behaviors of oneself and others (in this case the children) in terms of internal mental states, that is, thoughts, desires, and/or expectations. It can be performed in parents of children from 0 to 5 years old, and the results are obtained in three different dimensions or subscales: “prementalization” (PM), which involves an inability to hold the child’s mental states in mind and/or to have malevolent attributions about the child’s behavior; “certainty about mental states” (CMS), which refers to the acknowledgment that a parent’s thoughts about the child’s mental states are accurate; and “interest and curiosity” (IC), which involves an interest in parents thinking about their child’s mental states. In the development of the questionnaire, estimates of internal consistency (alpha coefficient) of 0.70, 0.82, and 0.75 were collected for the three dimensions, respectively. In the Spanish validation, values of 0.60, 0.77, and 0.66 were obtained, respectively [14]. In our sample, alpha coefficients were 0.57 for PM, 0.85 for CMS, and 0.63 for IC.

Experiences in Close Relationships-Revised [28]: This is a self-administered questionnaire of 36 items evaluated according to a 7-point Likert scale to assess adult attachment styles (1= strongly disagree to 7= strongly agree). Half of the items assess anxious attachment and the other half assess avoidant attachment. Anxious attachment is characterized by a fear of abandonment and anger at separation. Avoidance, on the other hand, is defined by a lack of closeness and emotional repression. The scale is highly reliable, with alpha coefficients of 0.90 for the anxiety scale and 0.91 for the avoidance scale [29], and a correlation between them of 0.41 [30]. The formulation of the items is such that it preferentially explores romantic attachment, providing the alternative of thinking of any other close relationship in the case of not having a partner. We used the Spanish validation of the questionnaire, which proved to have acceptable internal consistency (alpha coefficients of 0.83 and 0.86 for attachment anxiety and avoidance, respectively) and an interscale correlation of 0.18 [31]. Alpha coefficients in our sample were 0.86 for attachment anxiety and 0.78 for attachment avoidance.

### Statistical Analyses

First, a descriptive analysis was performed. As the results obtained in the different subscales did not follow a normal distribution (*p*-value of the Shapiro-Wilk test below 0.05 in all the scales except for Anxiety, which had a *p*-value of 0.067), we used non-parametric tests in the statistical analyses. Median and interquartile range of the results obtained with the different scales were calculated, both for the overall sample and stratifying by sex (mothers and fathers). The PRFQ score was also calculated in a disaggregated way, taking into account both the sex of the parents as well as the children (**Supplementary Table 1**). The sex distribution and mean age of each group were calculated. Subsequently, the Mann Whitney U test was used to determine whether there were differences in the results of the different scales based on the sex of the parents and children. Pearson’s correlation was used to analyze the relationship between quantitative variables (age of parents and children, and results on the different scales). Three univariate general linear models were created with the three PRFQ scales as dependent variables and with all those variables that had obtained a *p*-value <0.1 in the univariate analyses as independent variables. Bootstrapping (n = 1000) was applied using a simple resampling method, since it provides robust estimates when normality assumptions cannot be applied. Finally, to contrast results between groups of mothers and fathers, the same models were created in a disaggregated manner between mothers and fathers, excluding the sex variable of the parent in cases in which it had been included as an independent variable. Bootstrapping (n = 1000) was also applied in the disaggregated models.

## Results

There were significant differences between the group of mothers and fathers in the scores obtained across the different dimensions of the scales (Table 1). Regarding attachment style, the differences were not statistically significant, although, in the anxious attachment dimension, the fathers’ scores tended to be higher than those of the mothers. As regards PRFQ, there were significant differences in CMS and IC, with significantly higher scores obtained by mothers in both dimensions. There were no differences in any of the PRFQ dimensions when disaggregating the data by sex of the parent and children, that is, there appeared to be no differences in PRF depending on the sex of the child, neither in the group of fathers nor mothers (**Supplementary Table 1**). Table 2 shows several significant correlations, both positive and negative, with the PRFQ dimensions: anxiety and avoidance correlated with PM, anxiety with CMS, and avoidance with IC.

**Table 1.** **Medians, interquartile range and differences between fathers and mothers across the ECR-R and PRFQ dimensions**.

Variables		Median (Q1–Q3)	U	*p*
ECR-R Anxiety (N = 240)				
	Mothers	2.83 (2.22–3.55)	2466.50	0.091
	Fathers	3.11 (2.55–3.75)		
	All	2.86 (2.27–3.59)		
ECR-R Avoidance (N = 240)				
	Mothers	2.27 (1.94–2.73)	2811.00	0.502
	Fathers	2.33 (1.94–2.91)		
	All	2.33 (1.94–2.77)		
PRFQ PM (N = 262)				
	Mothers	1.50 (1.16–1.83)	3395.50	0.240
	Fathers	1.66 (1.16–1.87)		
	All	1.50 (1.16–1.83)		
PRFQ CMS (N = 262)				
	Mothers	3.66 (2.83–4.50)	4775.50	**0.029**
	Fathers	3.16 (1.83–4.08)		
	All	3.66 (2.66–4.50)		
PRFQ IC (N = 262)				
	Mothers	6.16 (5.66–6.66)	4885.50	**0.014**
	Fathers	5.83 (5.29–6.20)		
	All	6.16 (5.66–6.66)		

ECR-R, Experiences in Close Relationships-Revised; PRFQ, Parental Reflective Functioning Questionnaire; PM, Prementalization; CMS, Certainty about Mental States; IC, Interest and Curiosity. Significant results are in bold.

**Table 2.** **Spearman’s correlation between age, PRFQ and ECR-R (N = 262)**.

Variables	Parents’ age	Children age	PRFQ PM	PRFQ CMS	PRFQ IC	ECR-R Anx	ECR-R Avo
Parents age	-						
Children age	0.284**	-					
PRFQ PM	0.118^#^	0.066	-				
PRFQ CMS	–0.085	0.145*	–0.311**	-			
PRFQ IC	–0.070	0.119^#^	–0.153*	0.052	-		
ECR-R Anx	0.113^#^	–0.038	0.421**	–0.256**	–0.048	-	
ECR-R Avo	0.119^#^	0.012	0.211**	–0.075	–0.130*	0.268**	-

**p < 0.01, *p < 0.05, ^#^*p* < 0.10. PRFQ, Parental Reflective Functioning Questionnaire; PM, Prementalization; CMS, Certainty about Mental States; IC, Interest and Curiosity; ECR-R, Experiences in Close Relationships Revised; Anx, Anxiety; Avo, Avoidance. Significant results are in bold.

In the general linear models (Table 3), we observed that anxious attachment was associated with higher PM scores after controlling for avoidant attachment, which was no longer significantly associated with PM. The model explained 15.8% (adjusted R^2^ = 0.158) of PM variability. This association between anxious attachment and PM remained constant when performing sex-disaggregated analyses with the same model as a base.

**Table 3.** **General linear models with PRFQ dimensions as dependent variables and attachment, and parents’ and children’s age and sex as independent variables (N = 262). Bootstrapping (n = 1000) was applied**.

	Predictors	Bootstrapped β			95% BCa CI [inferior, superior]			*p*			Adj R^2^		
		M	F	All	M	F	All	M	F	All	M	F	All
1	Intercept	0.591	–0.783	0.426	–0.046, 1.172	–1.496, 0.036	–0.161, 1.024	0.067	0.062	0.149	0.124	0.392	0.158
	Parents age	0.006	0.032	0.009	–0.008, 0.023	0.002, 0.058	–0.004, 0.021	0.416	0.058	0.205			
	ECR-R Anx	0.224	0.353	0.240	0.138, 0.313	0.162, 0.692	0.156, 0.338	< **0.001**	**0.014**	< **0.001**			
	ECR-R Avo	0.049	0.033	0.059	-0.047, 0.165	–0.332, 0.337	–0.039, 0.160	0.391	0.763	0.248			
2	Intercept	4.219	4.357	3.890	3.561, 4.893	2.578, 5.926	3.145, 4.681	< **0.001**	< **0.001**	< **0.001**	0.041	0.138	0.070
	Children age	0.071	0.194	0.095	–0.028, 0.169	0.048, 0.478	0.004, 0.191	0.149	**0.033**	**0.038**			
	ECR-R Anx	–0.258	–0.501	–0.288	–0.442, –0.070	–1.085, 0.059	–0.456, –0.121	**0.004**	**0.043**	**0.002**			
	Parents sex (male)	-	-	0.354	-	-	–0.084, 0.813	-	-	0.137			
3	Intercept	6.297	5.598	5.922	5.882, 6.692	4.581, 6.994	5.484, 6.382	< **0.001**	< **0.001**	< **0.001**	0.010	0.026	0.038
	Children age	0.035	0.118	0.049	–0.018, 0.087	–0.038, 0.292	0.000, 0.103	0.238	0.066	**0.048**			
	ECR-R Avo	–0.110	–0.030	–0.098	–0.237, 0.025	–0.596, 0.347	–0.244, 0.029	0.102	0.880	0.146			
	Parents sex (male)	-	-	0.309	-	-	0.066, 0.558	-	-	**0.013**			

1, PRFQ Prementalization 2, PRFQ Certainty about Mental States 3, PRFQ Interest and Curiosity. PRFQ, Parental Reflective Functioning Questionnaire; ECR-R, Experiences in Close Relationships Revised; Anx, Anxiety; Avo, Avoidance; M, Mothers; F, Fathers. Significant results are in bold.

Anxious attachment also presented a significant association, in this case negative, with CM, although the variability explained by the model was much lower, 7% (adjusted R^2^ = 0.070, *p* < 0.001), than in the case of PM, suggesting that variables other than attachment type probably play a much more decisive role in explaining this aspect of PRF. Differences between the group of fathers and mothers were observed once again in the sex-disaggregated analyses, with a higher variability explained among fathers than mothers. It should be noted from this second model that both the age of the child and parent significantly influence this PRF dimension. Children age was also associated with greater CMS when the analysis was performed with the whole sample, and also when only fathers were evaluated, but it did not seem to affect mother’s CMS results.

Finally, in the IC model, the variables that seemed to have an independent and significant influence were the sex of the parent (men scored significantly lower) and the age of the child, observing that with older age of the child, the parents report greater interest and curiosity. Interestingly, this association did not hold when the analysis was disaggregated by gender, so this finding requires further analysis. Avoidant attachment, which presented a significant correlation in the univariate analysis, lacked any type of association when included in the model; this lack of association was consistent in the analyses disaggregated by sex.

## Discussion

### Adult Attachment and Mentalization

The use of hyperactivation and attachment deactivation strategies (associated with anxious and avoidant attachment, respectively) are related to impairments in reflective functioning and, in turn, give rise to poor mentalizing [3]. Secure parental attachment is thought to foster parental mentalizing, and in this sense, the strong association found between anxious attachment and a greater tendency to use prementalizing modes, as well as to hypermentalize (indicated by high CMS) is, therefore, plausible. The relationship between anxious attachment and PM of the PRF is also the most robust and consistent, in different works as well as between sexes in our sample.

Slade *et al*. [9], who assessed adult attachment with the AAI, criticized that categorical assessment makes it less precise when evaluating this association, while a continuous assessment, such as that provided, for example, by ECR-R, preserves degrees of difference among subjects. They found that insecure attachment categories consistently presented more maladaptive scores in PRF than the secure attachment category, and suggested the inability to envision mental states as something intrinsic to all categories of insecure attachment. The association found in our study, where higher scores in anxious attachment were associated with higher scores in PM, replicated this observation with continuous measures. It is plausible and coherent that anxious attachment, which reflects a tendency to pay exaggerated attention to distress and react with emotional hyperarousal, is transferred and probably amplified in the parent-child dyad, since it is a type of relationship that is naturally disrupted by high-intensity and frequently distressing affects. That is, we found that the tendency in close adult relationships to present anxious defenses when dealing with emotional and interpersonal stress, which intrinsically implies a difficulty to envision mental states, translates into a lesser ability to hold the child’s mental states in mind (high PM) and into a tendency to hypermentalize (high CMS). Both dimensions indicate difficulties in social cognition, and along with attachment patterns, they are primarily unconscious processes, stored as procedural memory, and heavily influenced by the adult’s own early experiences.

Attachment style has been considered a lifespan construct that guides thoughts, feelings, and behaviors in the interpersonal sphere “from the cradle to the grave” [32]. However, the evidence indicates that it is a dynamic disposition that is molded throughout the lifespan, which, although determined in large part by the first experiences with the attachment figures, is also influenced by changes in the relationship with these attachment figures (with both positive and negative valences), by peer experiences throughout life, as well as by the broader sociocultural context [33]. In this sense, using as a measure of adult attachment instruments such as the ECR-R, which assesses the current disposition towards hyper- or hypoactivation of attachment mechanisms in close relationships, is probably a measure that captures, in a more updated way, the current disposition also with respect to the child. Also, following the proposal by Jones *et al*. [34], we emphasize that the parent-child relationship is a component of a broader family system that includes both the romantic relationship of the parents and their ability to trust and feel comfortable in close relationships, hence the relevance of assessing adult attachment with self-reports . It is also important to highlight the “loose coupling” [35] cited by several authors on the relationship between PRF, parents’ attachment narratives, and parental emotional availability, considering that, although during childhood PRF might largely determine the mentalization of the child, other factors will shape this ability during the lifespan [4, 18]. Finally, and taking into account the small number of studies using this perspective of adult attachment, other authors have also stressed the need for more work examining parents’ subjective, self-reported attachment-related coping strategies in intimate adult relationships and their relation to parental mentalization [18].

### Influence of the Child’s Age and Parent’s Sex on CMS and IC

In our work, we observed that an older age of the child was associated with a higher score in both CI and CMS. Both models were controlled for the sex of the parent; however, when performing the models separately in mothers and fathers, we observed that this significant association only remained significant among fathers, with respect to CMS. It is striking that these correlations between the age of the child and the CMS and CI dimensions, which could be obvious in the light of the results of the entire group (greater knowledge and interaction capacity of the child translates into greater certainty about their mental states and also a greater interest in the child), cease to have weight when analyzing mothers and fathers separately. However, this lack of strength agrees more with the observations of the other published work that analyzed the relationship between the child’s age and PRF using the same instruments and a similar population (general, not clinical) [4]. We also found that mothers scored significantly higher than fathers in both CMS and IC, with no differences observed in the PM dimension, findings similar to those reported by other groups [4, 19]. It was suggested that these differences in CMS and CI between mothers and fathers could reflect differences in the intensity of maternal and paternal care [4], citing the “primary maternal preoccupation” [36], which is typically more pronounced among mothers, also in our environment. Finally, we must bear in mind that the authors of the PRFQ pointed out that, for both IC and CMS, average scores would be the healthiest. In the CMS dimension, high scores would reflect a difficulty in assuming the opacity of the child’s mental states and hypermentalization and, in the IC dimension, intrusiveness. The scores of our sample, which as we have already pointed out was from the general population, were similar to those observed both in the general population sample reported by the developers of the PRFQ [4], and those of the Spanish validation study [14], also carried out in the general population. It should be noted, regarding the idea of the normativity of average scores, that, for the CMS dimension, the average scores of the three studies were at that midpoint (around 3.5 on a scale of 1 to 7). Nonetheless, for IC, most of the published works using PRFQ present average scores approaching six, so perhaps it would be necessary to rethink whether the IC captured by the items are truly a reflection of intrusiveness and hypermentalization, or a genuine and healthy interest, as we dare to defend.

### Effects of Parental Attachment and PRF on Child Development: Clinical Implications

As already mentioned in the introduction, PRF appears to relate to different aspects of child development. One of the most interesting proposals is that of PRF as a mediator of the intergenerational transmission of attachment [9, 10]. Specifically, Slade *et al*. [9], based on empirical work with robust theoretical justification, proposed PRF as the element that remains to be defined in the attachment transmission gap. They assessed the attachment style and PRF in parents and the attachment style in children. They observed powerful relationships between maternal RF and infant attachment security, and they also performed an exploratory analysis revealing that maternal RF fully mediated the association between the attachment of the mother and the infant. Based on the latter finding, they proposed PRF as that missing element of the transmission gap. The predictive capacity of PRF for the quality of attachment in infants has subsequently been replicated in other studies [4, 17, 37], also using different tools to assess both PRF and attachment in infants. There is only one study published on the association between PRF and attachment in offspring that has not found an association [13]. However, as we previously mentioned, they used mind-mindedness to assess PRF, a test that captures aspects that are more behavioral and apparently different from those measured by the other tests that assess PRF. RF, without being specifically PRF (that is, evaluating the RF scale in the AAI), has also been related to more secure attachment and better RF in the offspring [6, 37].

Besides, PRF seems to affect other aspects of the child’s socio-emotional development [38] beyond mentalization and attachment [38, 39]. Nijjssens *et al*. [38], pointed out that impairments in PRF are negatively related to the child’s socio-emotional development, and that high levels in prementalizing modes mediate the relationship between avoidant and anxious attachment styles and child development. PRF has also been associated with quality of care (reviewed in [38]) and parental behaviors [40], and has been described to mediate maternal and offspring psychopathology [41], infant socio-emotional development [42], and the association between adult attachment style and parenting satisfaction [43]. Probably, the relationship between all these aspects of the child’s development is produced through interwoven and multidirectional mediating and moderating effects that will have to be unraveled in future research given the relevance they may have as targets for preventive interventions. From a perspective of mentalization as a transtheoretical and transdiagnostic concept to explain vulnerability to psychopathology [3], both preventive intervention in parents and early detection and intervention in the offspring with imbalances in the quality of mentalizing could have a preventive effect on the future development of mental disorders. There are manual interventions to improve RF specifically aimed at parents [39], which could be used with subjects identified as at risk, as well as interventions to enhance mentalizing in children; both types of interventions could be implemented as strategies for promoting children’s mental health and preventing psychopathology.

### Strengths, Limitations, and Proposals for Future Lines of Research

This work aimed, on the one hand, to study the association between adult attachment and PRF using self-reports and, on the other, to try to replicate the scarce works published on the subject incorporating a sex-sensitive approach. Its main strengths are the large sample size and, above all, the tools used, which have proven their validity in evaluating the psychic dimensions they intend to measure. These are accessible and also stand out for their simplicity of completion, favoring the possibility of using them in routine clinical practice, or even as screening tools in large populations, facilitating the detection of subjects who could benefit from specific interventions. As a main limitation, we would highlight that the father subgroup was small, thus results concerning this subgroup of parents must be assumed as only exploratory. Other important limitations, which could also serve as proposals for new lines of research, would be not having collected other types of clinical and sociodemographic data that could influence the dimensions studied, such as the educational and socio-economic levels, or the assessment of other psychic variables such as intrusion, parental competence, or a general psychopathology scale. Likewise, it would have been interesting to complete the study with the assessment of attachment in children, although we are once again faced with the difficulty involved in assessment standards today, based on observational examinations.

This work, therefore, on the one hand, replicates previous findings regarding the relationship between dysfunctional dimensions of attachment and PRF in parents and, on the other, discusses the long-term implications that the quality of PRF seems to have in the offspring. If we consider the impact that it seems to have on the development of the child’s socio-emotional competencies (including their own mentalization) as well as their possible role as an intergenerational transmitter of dysfunctional (or functional) patterns of attachment, it is truly relevant that we have simple and generalizable measures to assess PRF on a large scale, such as the PRFQ. Having evidence indicating that adult attachment style, which can also be assessed by simple self-reported scales, predicts the quality of PRF also puts us in a position to detect candidates who could benefit from attachment-based or mentalization-based interventions in a prenatal or even preconception period, which would have the potential to benefit both the parent and the future offspring.

## Conclusions

The results of this study confirm, on the one hand, the strong relationship between insecure attachment (mainly anxious) and poor PRF, marked by prementalizing modes and hypermentalization. On the other hand, they highlight differences in PRF and between mothers and fathers, as well as differences in how children’s age affect PRF, suggesting that mothers present a greater reflective capacity in some areas, and are less influenced by children’s age. Finally, it supports the use of self-reports to assess PRF, which could facilitate its extensive evaluation in the target population.

## Availability of Data and Materials

The datasets used and/or analyzed during the current study are available from the corresponding author on reasonable request.

## Supplementary Material

Supplementary material associated with this article can be found, in the online version, at https://doi.org/10.62641/aep.v54i4.2080.
