# Supplementary material for: Adult Romantic Attachment and Parental Reflective Functioning in Parents of Pre-School Children: An Association Study With a Sex-Sensitive Approach
Source: Actas Esp Psiquiatr. 2026 Aug 15;54(4):1234–44. doi: 10.62641/aep.v54i4.2080 (PMC13571180; doi:10.62641/aep.v54i4.2080)
Supplement: Supplementary file 1 [file ActEsp-54-4-1234-1244-s1.zip › Supplementary Table 1.docx]

**Supplementary Table 1. Mean differences in the score obtained in the PRFQ subscales according to the gender of parents and offspring**

|  | PRFQ dimension | Girls^#^  Mean±SD | Boys^#^  Mean±SD | U | p |
| --- | --- | --- | --- | --- | --- |
| Mothers | PM | 1.56 ± 0.491 | 1.57 ± 0.670 | 6601.00 | 0.522 |
| N = 225 | CM | 3.71 ± 1.228 | 3.64 ± 1.134 | 6579.50 | 0.554 |
|  | IC | 6.08 ± .631 | 6.15 ± 0.660 | 5755.50 | 0.269 |
| Fathers | PM | 1.73 ± .576 | 1.63 ± 0.589 | 159.50 | 0.560 |
| N = 33 | CM | 2.94 ± 1.206 | 3.29 ± 1.490 | 134.50 | 0.784 |
|  | IC | 5.90 ± .770 | 5.57 ± 0.662 | 114.50 | 0.336 |

PRFQ: Parental Reflective Functioning Questionnaire; SD: Standard Deviation; PM: Prementalization; CM: Certainty about Mental States; IC: Interest and Curiosity

\# Mothers: Girls N=121, Boys N=104. Fathers: Girls N=14, Boys N=19
